# Altered functional resting-state hypothalamic connectivity and abnormal pituitary morphology in children with Prader-Willi syndrome

**DOI:** 10.1186/s11689-017-9188-7

**Published:** 2017-02-21

**Authors:** Akvile Lukoshe, Suzanne E. van Dijk, Gerbrich E. van den Bosch, Aad van der Lugt, Tonya White, Anita C. Hokken-Koelega

**Affiliations:** 1 Dutch Growth Research Foundation, Postbus 23068, 3001 KB Rotterdam, The Netherlands; 2 Department of Pediatrics, Erasmus MC-Sophia Children’s Hospital, Postbus 2060, 3000 CB Rotterdam, The Netherlands; 3 Department of Child and Adolescent Psychiatry, Erasmus MC-Sophia Children’s Hospital, Postbus 2060, 3000 CB Rotterdam, The Netherlands; 4 Intensive Care and department of pediatric surgery, Erasmus MC-Sophia Children’s Hospital, Postbus 2060, 3000 CB Rotterdam, The Netherlands; 5 Department of Radiology, Erasmus Medical Centre-Sophia Children’s Hospital, Postbus 2040, 3000 CA Rotterdam, The Netherlands

**Keywords:** Prader-Willi syndrome, 15q11-q13, Functional resting-state connectivity, Hypothalamus, Pituitary gland, Neurodevelopmental disorders

## Abstract

**Background:**

Prader-Willi syndrome (PWS) is a complex neurodevelopmental disorder, characterized by endocrine problems and hyperphagia, indicating hypothalamic-pituitary dysfunction. However, few studies have explored the underlying neurobiology of the hypothalamus and its functional connectivity with other brain regions. Thus, the aim of this study was to examine the anatomical differences of the hypothalamus, mammillary bodies, and pituitary gland as well as resting state functional connectivity of the hypothalamus in children with PWS.

**Methods:**

Twenty-seven children with PWS (13 DEL, 14 mUPD) and 28 typically developing children were included. Manual segmentations by a blinded investigator were performed to determine the volumes of the hypothalamus, mammillary bodies, and pituitary gland. In addition, brain-wide functional connectivity analysis was performed using the obtained masks of the hypothalamus.

**Results:**

Children with PWS showed altered resting state functional connectivity between hypothalamus and right and left lateral occipital complex, compared to healthy controls. In addition, children with PWS had on average a 50% smaller pituitary volume, an irregular shape of the pituitary, and a longer pituitary stalk. Pituitary volume did not increase in volume during puberty in PWS. No volumetric differences in the hypothalamus and mammillary bodies were found. In all subjects, the posterior pituitary bright spot was observed.

**Conclusions:**

We report altered functional hypothalamic connectivity with lateral occipital complexes in both hemispheres, which are implicated in response to food and reward system, and absence of connectivity might therefore at least partially contribute to the preoccupation with food in PWS.

## Background

Prader-Willi Syndrome (PWS) is a rare and poorly understood neurodevelopmental disorder that affects 1 in 15,000 live births. PWS is caused by the lack of expression of the paternally derived genes on chromosome 15 at locus q11-q13, due to a deletion (DEL [1], maternal uniparental disomy (mUPD [2]), unbalanced translocation, or imprinting center defect [3]). PWS is characterized by hyperphagia, which is a major concern for patients with PWS and their caregivers, as it may lead to obesity, diabetes mellitus, cardiovascular diseases, and, in some cases, death due to stomach rupture. In addition, patients with PWS suffer from developmental delays and multiple abnormalities of the endocrine system, such as short stature, hypogonadism, delayed and incomplete sexual maturation and infertility [4, 5], high pain threshold, and abnormalities in temperature regulation.

Clinical studies have yielded substantial evidence for a hypothalamus-pituitary axis dysfunction in PWS. Children with PWS have low insulin-like growth factor I (IGF-I) levels prior to treatment with growth hormone (GH) [6] and might have decreased GH secretion after provocative testing [7], which contributes to short stature, increased fat mass, and decreased lean body mass in PWS. Furthermore, irregular or absent menstrual cycle in girls, cryptorchidism, and infertility in boys, reduced secondary sexual characteristics, and decreased bone density are present in the vast majority of PWS patients, due to hypogonadism of both central and primary origin [4, 6]. Both normal and impaired functioning of hypothalamic-pituitary-adrenal (HPA) and thyroid axes have been reported, likely due to methodological differences [8–10].

Neurobiological mechanisms underlying endocrine problems and particularly hyperphagia in PWS are not yet fully understood. Smaller anterior pituitary [11], absent posterior pituitary bright spot [12], and pituitary empty sella [13] were found in adults with PWS, as well as a reduction in pituitary height in children with PWS [14], which might contribute to endocrine dysfunction in PWS. An increased functional response of the hypothalamus to food stimuli was found in functional brain imaging studies in PWS adults [15, 16]. These results, combined with studies showing structural abnormalities of the hypothalamus in PWS [17], suggest that a dysfunctional hypothalamus might underlie abnormal satiety regulation in PWS.

The major shortcoming of task-dependent functional magnetic resonance imaging (fMRI) studies is that the research outcome is dependent on the participant’s ability to understand the instructions of the task, posing a potential problem in patient populations with cognitive impairments. An alternate approach to functional brain activity in patients with PWS is to study resting state functional connectivity. Functional connectivity of the brain follows the principle of “what wires together, fires together” and allows for the quantification of how anatomically separated brain regions co-vary together while the brain is at rest, thus in the absence of any cognitive task. However, to date, there are limited studies performed in adults or children with PWS assessing global hypothalamic connectivity specifically. One study has reported increased connectivity between hypothalamus and amygdala in untreated children with PWS [18]. In an obese, but otherwise healthy cohort, both increased and decreased resting-state connectivities have been found in multiple brain areas responsible for behavioral control, reward and motivation [19, 20]. An abnormal functional response of the hypothalamus to food stimuli was found, indicating that hypothalamic dysfunction might underlie hyperphagia in PWS [15]. It is therefore likely that aberrant functional resting-state connectivity might underlie abnormal response to food not only in obese individuals but also in individuals with PWS.

Based on the neuroendocrine symptoms found in PWS, the primary goal of this study was to investigate the neurobiology of the hypothalamus, including resting state functional connectivity and volumetric measures of the hypothalamus, mammillary bodies (due to their distinct structure), and the pituitary gland in children with PWS compared to controls. Given the evidence of abnormal hypothalamic functioning in the PWS, we hypothesized that abnormal resting-state connectivity of the hypothalamus would be present in children with PWS, particularly with the brain areas implicated in reward and behavioral regulation. We also hypothesized that the hypothalamus, together with the pituitary gland, would be smaller in volume in children with PWS, compared to healthy controls, given the abundance of evidence for hypothalamus-pituitary dysfunction in PWS. In addition, we also measured the length of the pituitary stalk.

## Methods

### Participants

Twenty-seven children with PWS and 28 typically developing children were included. Inclusion criteria for PWS group were (1) genetically confirmed PWS, (2) age 6–23 years, and (3) no neurological or psychiatric history. Inclusion criteria for the healthy control group were (1) age 6–23 years, and (2) no neurological or psychiatric history. All children with PWS participated in the Dutch PWS Cohort Study [21]. The study was approved by the Medical Ethics Committee of the Erasmus Medical Center Rotterdam, Rotterdam, The Netherlands. Written informed consent was obtained in all cases from the caregivers and children older than 12 years and informed assent in children younger than 12 years.

### MRI acquisition

Children were introduced to a mock scanner before they underwent the MRI scan. Caregivers were given the option to stay in the room with the MRI scanner, close to their child.

All images were acquired on a 3T GE 750 Discovery MRI scanner (General Electric, Milwaukee, WI, USA), using a dedicated 8-channel head coil. Following 3-plane localizing and coil intensity calibration scans, a high-resolution T1-weighted inversion recovery fast spoiled gradient recalled (IR FSPGR) sequence was obtained with the following parameters: TR = 10.3 ms, TE = 4.2 ms, TI = 350 ms, NEX = 1, flip angle = 16°, readout bandwidth = 20.8 kHz, matrix 256 × 256, imaging acceleration factor of 2, and an isotropic resolution of 0.9 × 0.9 × 0.9 mm^3^ (duration; 5 min 40 s). Resting-state fMRI (rs-fMRI) utilized a gradient-echo blood oxygen level dependent (BOLD) EPI sequence with a TR = 2000 ms, TE = 30 ms, flip angle = 85°, matrix 64 × 64, and voxel resolution of 3.6 ×3.6 × 4.0 mm^3^. The duration of the rs-fMRI was 160 TRs (5 min 20 s). The children were asked to keep their eyes closed during the rs-fMRI sequence and to think about nothing in particular. All MRI images were reviewed by a qualified radiologist and no gross brain abnormalities were identified.

Twenty-two children with PWS and 27 healthy controls had both structural MRI and resting state fMRI data available, suitable for manual segmentation functional connectivity analysis, respectively.

### Data processing

#### Manual segmentation

All MRI images were coded to ensure investigators’ blindness to subject identification and diagnosis. Manual segmentation of the hypothalamus, mammillary bodies, and pituitary gland were performed with the FreeSurfer 5.3 image analysis suite. Tracing was performed slice by slice (1-mm thick) in coronal, axial, and sagittal orientations. Thus, the anatomic boundaries were viewed from different orientations. The hypothalamus segmentation boundaries used were based on the boundaries used by Klomp et al. [21] and Lemaire et al. [22]. The anterior boundary of the hypothalamus was defined by the first coronal slice after the anterior commissure (AC) and the posterior boundary by the last slice where the mammillary bodies were visible. The dorsal boundary consisted of the AC-PC (posterior commissure) plane in the transverse section or the hypothalamic sulcus and ventral border of the lamina terminalis. The lateral boundaries were determined by the white matter bundles. The boundaries of the mammillary bodies were defined anterior by the first coronal slice in which the mammillary bodies were visible as part of the hypothalamus and posterior by the very last slice in which they were still visible. The floor of the third ventricle and the cerebral spinal fluid (CSF)-filled suprasellar cistern defined the dorsal and ventral boundaries, respectively. Laterally the boundaries consisted of the darker gray of the hypothalamus or the bundle of white matter that surrounds it.

We measured pituitary volume instead of pituitary height, as the latter measure only weakly correlates with pituitary volume [23]. The boundaries of the pituitary gland were defined anteriorly and ventrally by the sphenoid sinus, posteriorly by the dorsum sellae, dorsally by the diaphragma sellae, and laterally by the cavernous sinuses. The infundibular stalk was excluded from the segmentations. For each subject, it was assessed whether the posterior pituitary bright spot (PPBS) was present. The length of the pituitary stalk was defined and measured as the distance from the tip of the infundibular recesses to the junction of the pituitary stalk and the pituitary gland, along the course of the pituitary stalk.

#### Resting-state functional connectivity

All rs-fMRI data were preprocessed by the FMRIB’s Diffusion Toolbox (FDT) from the FMRIB’s freely available Software Library (FSL, http://www.fmrib.ox.ac.uk/fsl). First, the first four volumes of rs-fMRI scan of each participant were discarded to allow for T1-equilibration effects. Each subject’s functional images were motion-corrected with MCFLIRT, spatially smoothed with an 8-mm spatial filter (White et al. 2001) and denoised using FSL’s ICA-based X-noiseifier (FIX) tool [24], regressing out six motion parameters. Individual hypothalamus masks, obtained from the manual segmentations, were registered into functional space using FLIRT, and the individual fMRI time series within the hypothalamus ROI were extracted. Individual whole-brain voxel-wise regression analyses were performed with FSL’s FEAT using the time series within the hypothalamus as the independent variable, controlling for the white matter and CSF time series. The obtained z-transformed individual images were then registered to the high-resolution structural image, and both structural and functional images were registered into standard space using the Montreal Neurological Institute T1 template (MNI52) using FSL’s FLIRT. We then generated group mean connectivity maps as well as tested for group differences (PWS vs. control, DEL vs. control, mUPD vs. control and DEL vs. mUPD), using *FSL*’*s* randomize permutation-testing tool with 5000 permutations, excluding white matter and CSF voxels, and threshold-free cluster enhancement (TFCE), accounting for age and gender effects and setting *p* value at <0.05. The minimal number of voxels for a cluster to be considered was set at 50. Family-wise error (FWE) was used to correct for multiple comparisons. Fisher’s z-transformed partial correlation coefficients were estimated.

### Hormones and assays

Blood samples for the assessment of IGF-1, thyroid stimulating hormone (TSH), T4, free T4 (fT4), T3, and reverse T3 (rT3) were collected prior to start with GH treatment, in the morning, after overnight fasting. After centrifugation, the samples were immediately frozen at –20 °C until assayed.

Serum IGF-I levels were measured in one central laboratory using a immunometric technique on an Advantage Automatic Chemiluminescence System (Nichols Institute Diagnostics, San Juan Capistrano, CA). The intraassay coefficient of variation was 4%, and the interassay coefficient of variation was 6%. Because of age and sex dependency, IGF-I levels were transformed into standard deviation scores (SDS) [25].

Plasma levels of rT3 were measured by established radioimmunoassay procedures, as described previously [10]. Serum TSH, free T4, T4, and T3 levels were measured by Vitros Eci technology (Ortho-Clinical Diagnostics, Amersham, UK) [10]. The within-assay coefficient of variation (CV) was 3–7% and the between-assay CV 5–10%. Thyroid hormone SDSs were calculated with data from a control group comprising 500 healthy Dutch children (aged 1.4–18 years) from the Rotterdam region, The Netherlands, participating in a study to assess reference values.

The fasting blood samples were taken for the analysis of maximal adrenocorticotropic hormone (ACTH) levels after overnight single-dose metyrapone test, after start of treatment with GH (for detailed description, see [8]). Plasma ACTH levels were measured with an immunoradiometric assay (Bio-International, Gif-sur-Yvette, France) with a minimal detection level of 1.1 pmol/liter. Glucose levels were measured with the Hitachi 917 (Hitachi Device Development Center, Tokyo, Japan), detecting glucose levels between 0 and 42 mmol/liter.

### Statistical analyses of volumetric data

Intrarater reliability of the volume measurements, assessed by intraclass correlation coefficient (ICC) in the five brains was as follows: the hypothalamus (ICC = 0.97), mammillary bodies (ICC = 0.73), and pituitary gland (ICC = 0.99).

Interrater reliability of the volume measurements, assessed by intraclass correlation coefficient (ICC), for five randomly selected patients by two raters (AL and SD) who were both blinded to subject identification and diagnosis was as follows: the hypothalamus (ICC = 0.93), mammillary bodies (ICC = 0.76), and pituitary gland (ICC = 0.99).

Volumes of the hypothalamus, mammillary bodies, and pituitary gland and pituitary stalk length were exported to SPSS (version 21, IBM Corporation, Armonk, NY, USA) for statistical analyses. Groups were compared using a nonparametric Mann-Whitney or Kruskal-Wallis analysis of variance test and a chi-square, where appropriate. For significant group effects only, post hoc Mann-Whitney tests were performed for pairwise comparisons with a Bonferroni correction of *p* = .05/3 (DEL, mUPD, and control). For interaction effects between pituitary volume and age, nonparametric Quade’s rank analysis of covariance was performed.

## Results

Demographic data and clinical data of the patients are presented in Table 1. No significant differences were found in age and gender distribution between both groups.

**Table 1:** Demographic and clinical data of the participants

	PWS		Control	*p* value	Correlations with pituitary gland (in PWS only)	
	DEL	mUPD			rho	*p* value
Sample size (*n*)	14	13	28			
Age (years)	13.7 (3.5)	12.5 (5.1)	13.0 (3.1)	.93		
Age range (years)	8.1–21.4	6.0–23.3	7.1–18.0			
Male (*n*)	6	3	11			
Female (*n*)	8	10	17			
IGF-I SDS	−2.0 (1.5)	−3.2 (1.9)		.04	.31	.14
TSH (mU/l)	2.2 (1.1)	2.4 (1.0)		.62	.51	.016
Free T4 (pmol/l)	16.6 (2.5)	16.2 (2.3)		.43	−.22	.32
T3 (nmol/l)	2.6 (.7)	3.2 (1.9)		.25	.11	.64
Reverse T3 (nmol/l)	.37 (0.6)	.38 (1.0)		.97	−.49	.03
ACTH (pmol/l)	30.2 (20.1)	30.3 (17.6)		1.0	−.36	.23

Data expressed as mean (SD) or number. No significant differences were found in either age or gender distribution between the groups

*DEL* deletion, *mUPD* maternal uniparental disomy, *PWS* Prader-Willi syndrome, *SD* standard deviation

### Hypothalamic functional connectivity

**Fig. 1:**
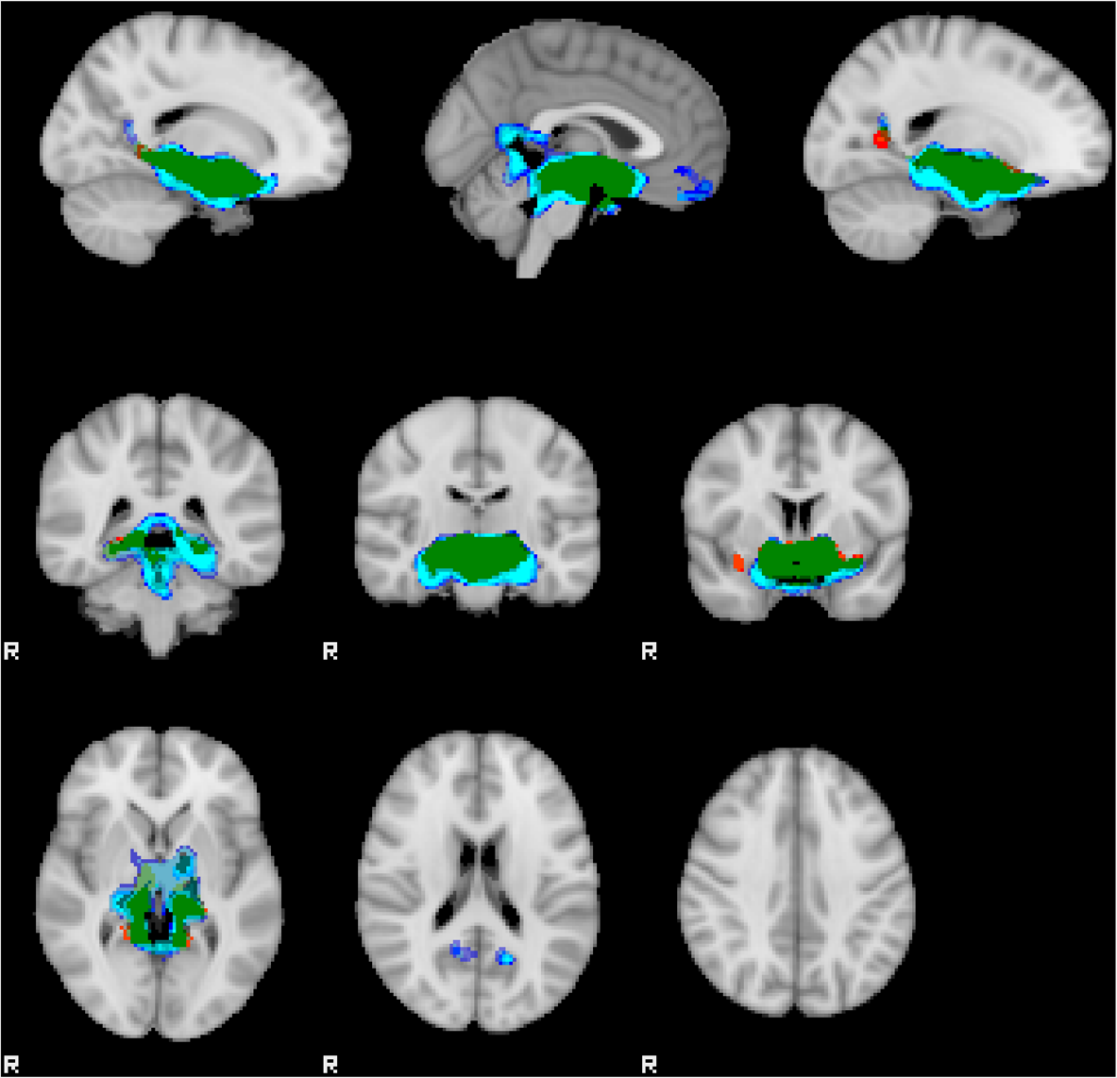
Hypothalamic connectivity maps in individuals with PWS and healthy controls. Hypothalamic connectivity maps: *blue*, healthy controls; *red*, PWS; *green*, overlapping connectivity. Connectivity maps were processed with TFCE and thresholded at FWE-corrected *p* < .05

**Fig. 2:**
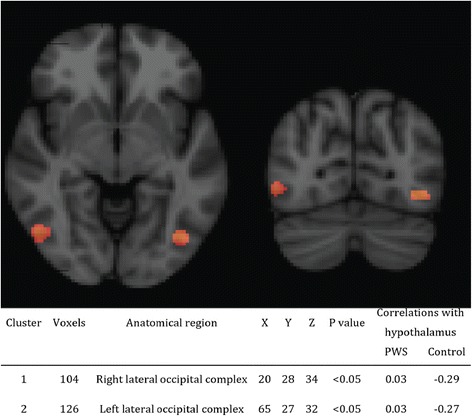
Altered functional connectivity between the hypothalamus and lateral occipital cortex in PWS. The coordinates, size, corresponding partial correlation coefficients, and *p* values are indicated underneath in Fig. 2

Hypothalamic connectivity maps for PWS and controls are presented in Fig. 1. After accounting for multiple testing, children with PWS, compared to healthy controls had increased connectivity between the hypothalamus and two small clusters in both the right (104 voxels, *p* < .05) and left (126 voxels, *p* < .05) lateral occipital complex (LOC) (Fig. 2). Fisher’s z-transformed partial correlations between the hypothalamus and lateral occipital complexes were negative in controls and close to 0 in PWS (Fig. 2). No other brain areas were found to have altered functional connectivity, either increased or decreased, with the hypothalamus. No significant differences were found between genetic subtypes, and no trends were detected either.

### Volumes of the hypothalamus and mammillary bodies

There were no significant differences in volume of the hypothalamus or mammillary bodies between children with PWS and healthy controls (Table 2). During the manual tracing, no anatomical aberrations were observed, the hypothalamus and mammillary bodies had normal appearance in all subjects.

**Table 2:** Hypothalamus and pituitary gland volumes in children with PWS

	PWS				Control		
	mUPD		DEL				
	Mean	SD	Mean	SD	Mean	SD	*p* value^a^
Hypothalamus	5395.0	1109.5	5542.2	1101.6	5657.3	1349.3	.82
Mammillary bodies	922.8	177.5	788.8	85.7	970.1	168.8	.33
Pituitary gland	1279.4	501.4	1267.3	394.0	2674.6	1057.8	<.0001
Posterior pituitary bright spot	13/13		14/14		28/28		
Pituitary stalk length (mm)	7.9	1.9	7.07	2.5	5.6	2.0	.041
Interrupted stalk	0/13		0/14		0/28		

Data expressed as mean (SD) or number (mm^3^) unless indicated otherwise. No significant differences were found in either age or gender distribution between the groups

*DEL* deletion, *mUPD* maternal uniparental disomy, *PWS* Prader-Willi syndrome, *SD* standard deviation

^a^PWS vs. control

### Volume of pituitary gland

**Fig. 3:**
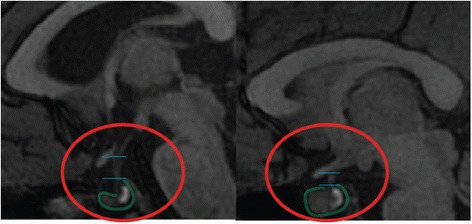
Pituitary gland abnormalities in PWS. *Left*, patient with PWS; *right*, healthy control; both are of the same age and gender. Note the present posterior *bright spot in both the left and right images*, within the *red circle*. The contours of the pituitary gland are marked with *green line. Horizontal blue lines* indicate the approximate location of the superior pituitary border and infundibular recess for the measurement of the pituitary stalk length. Please note the boat-like cavity around the place where pituitary stalk roots itself into the gland and a long pituitary stalk in PWS

**Fig. 4:**
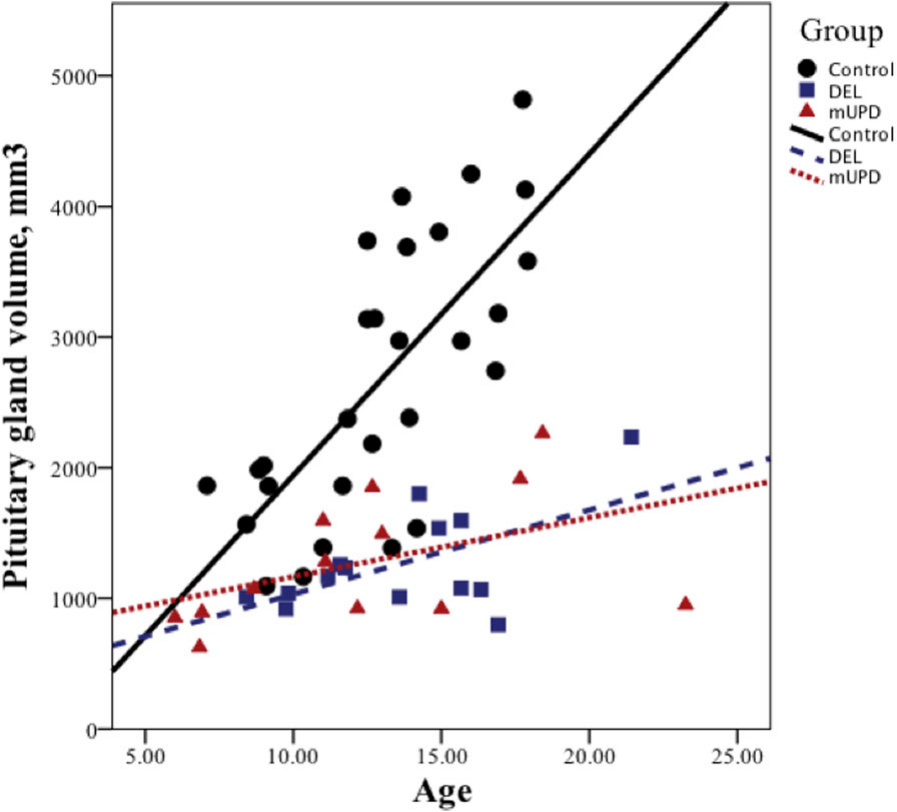
Pituitary volume and age in children with PWS and healthy controls. Pituitary volume was significantly different in both groups (*p* < .0001), with a significant pituitary volume by age interaction between PWS and controls (*p* < .0001)

A posterior pituitary bright spot (PPBS) was identified in all subjects (Fig. 3) The pituitary gland was smaller in the PWS children and had a flattened, boat-like shape, in combination with a longer pituitary stalk. The pituitary stalk was intact in all subjects. Children with PWS had a significantly a smaller pituitary volume (*p* < .001) and a significantly longer pituitary stalk (*p <* .05) compared to the healthy controls. Pituitary volume correlated strongly with age in both groups (rho = .40, *p* < .01 (combined); rho = .69, *p* < .0001 (control); rho = .46, *p* < .05 (PWS)). It did not increase in volume with age in PWS, in contrast with healthy controls (*p* < .0001). Post hoc analyses revealed no significant differences between the DEL and mUPD subtypes (Fig. 4).

### Pituitary gland volume correlations with baseline hormonal levels

Pituitary gland volume correlated positively with baseline TSH (*p* < .05) and showed a trend towards a positive correlation with baseline IGF-I SDS levels, but not with fT4 and ACTH (Table 1).

## Discussion

Our study investigated global functional resting-state connectivity as well as volume of the hypothalamus and pituitary gland in children with PWS. Children with PWS had no resting-state functional connectivity between the hypothalamus and lateral occipital complex (LOC) in both hemispheres, compared to negative correlation found in healthy controls. Hypothalamic volume was similar in both PWS and controls. In addition, children with PWS had a much smaller pituitary gland (on average 50% smaller), together with a longer, albeit uninterrupted pituitary stalk. PPBS was present in all cases, both in patients and controls.

The lateral occipital complex (LOC) is involved in visual processing, object recognition [26], and tracking the caloric value of foods [27]. In a recently conducted meta-analysis analyzing functional MRI studies that addressed processing of food vs. nonfood pictures, LOC appeared to be one of the most robust brain areas associated with processing food related stimuli, found in multiple studies, along with the orbitofrontal cortex and insula [28]. LOC is an important hub for processing of visual food information as it is functionally connected to the satiety center (hypothalamus), reward system (nucleus accumbens), and cognitive control center (orbitofrontal cortex) [29]. Visual network, including LOC, is also modulated by the limbic system, which results in the enhanced visual processing of emotionally relevant stimuli, including food [30]. It is therefore very intriguing that we found the absence of resting-state functional connectivity between LOC and the hypothalamus in individuals with PWS, which could potentially lead to abnormal processing of food cues and integration of satiety responses and thus be associated with the preoccupation with food seen in PWS patients. Interestingly, we did not find involvement of prefrontal cortex, in contrast to previous studies, which suggested an impairment in cognitive control mechanisms [31].

Although our findings may suggest that altered hypothalamic connectivity in PWS may relate to a preoccupation with food, thus contributing to hyperphagia, the autonomic symptoms of PWS of strictly hypothalamic origin (such as high pain threshold, inability to develop fever in case of illness), and their neurocorrelates remain to be clarified. Hypothalamus connectivity maps in our study largely resemble those obtained in another study, minor differences between the maps can be explained by methodogical aspects such as size of a chosen seed [29]. High-resolution microscopic studies or animal models might be necessary to further explain the hypothalamic impairment in PWS.

Surprisingly, no significant differences were found in the volume of the hypothalamus or mammillary bodies between PWS and healthy controls. Post mortem PWS studies showed that the size of paraventricular nucleus (PVN) and the number of oxytocin secreting neurons within PVN were reduced in patients with PWS [17]. As PVN is located within the medial part of the hypothalamus, it is possible that structural abnormalities, if any, are restricted to the medial hypothalamus only and are not observable when quantifying the entire hypothalamic volume. Due to lack of visible boundaries on the MRI images between hypothalamic nuclei, it is not possible with the current resolution found in clinical MRI machines to study these areas separately. It is important to note that post mortem studies were based on adults with PWS; therefore, we cannot exclude that some changes in the volume of the hypothalamus might have occurred later in life or after death.

The pituitary gland was significantly smaller in individuals with PWS and often had a flat shape, resembling a boat with a cavity-like opening encircling the region where the pituitary stalk roots itself into the gland, rather than a round pea-shaped structure as one would expect. We found PPBS to be present in all cases, both PWS and controls, in contrast to another study that reported the absence of the PPBS in patients with PWS [32]. Although our investigators were blinded for the diagnosis of the subjects, often the differences in the shape of the pituitary gland and the length of the stalk were so striking that they divided the study group in two subgroups—those with normally looking pituitary and a short stalk and those with a small pituitary and long pituitary stalk.

The volume of pituitary gland correlated strongly with age in both groups, although it did not show the same age-related increase during the pubertal period seen in the older adolescents and young adults with PWS. We did not assess pubertal stage in healthy controls as our study was not designed to address these issues, so we could not perform statistical analyses to support this observation. Based on our results, we can only speculate that in prepubertal children with PWS, the developmental differences seen in the pituitary gland may parallel the endocrine differences in children with PWS. Thus, one possibility is that the activation of the pituitary gland during puberty falls behind in the PWS children, resulting in inability to function optimally during puberty, which contributes to delayed growth and sexual maturation in individuals with PWS.

The lower pituitary volumes in the PWS group correlated with the lower TSH levels and showed a trend towards significance with the lower baseline IGF-I SDS levels, but not with the other baseline endocrine parameters. Children with PWS have reduced IGF-I levels and might also have low thyroid functioning [10, 25]. In addition, children with PWS had a significantly longer pituitary stalk compared to healthy controls. To our knowledge, a longer pituitary stalk has not been previously described. During the pituitary development, pituitary stalk forms from the neuroderm, together with posterior pituitary, and consists of axons that carry vasopressin and oxytocin and the releasing hormones from the medial hypothalamus to the pituitary gland [17]. It might be that connectivity between the hypothalamus and pituitary is adversely affected; however, due to susceptibility artifacts, the white matter connectivity between the pituitary and hypothalamus could not be measured.

There are several limitations to our study. First, the sample size was relatively small, although respectable for a study of PWS. A larger sample size would allow us to better evaluate differences between the genetic subtypes as well as the association with pubertal measures. Second, the cross-sectional design does not allow us to evaluate developmental trajectories in children with PWS compared to controls. Finally, at the time of the study, all children with PWS received GH treatment (1 mg/m^2^ per day); therefore, the children with PWS may have less abnormalities compared to untreated patients.

## Conclusions

Children with PWS showed altered resting state functional connectivity between the hypothalamus and LOC in both hemispheres compared to healthy controls. Given the involvement of LOC in the visual processing of food stimuli, the altered functional connectivity between the hypothalamus and LOC might at least partially underlie the preoccupation with food in PWS. No volumetric differences in the hypothalamus and mammillary bodies were found between PWS and controls. In contrast, children with PWS had on average a 50% smaller pituitary volume, an irregular shape of the pituitary, and a longer pituitary stalk. In all subjects, a posterior pituitary bright spot was observed. No significant differences were found between the DEL and mUPD subtypes in the morphology or connectivity of the hypothalamus.

## Abbreviations

(f)MRI: (Functional) magnetic resonance imaging
AC: Anterior commissure
ACTH: Adrenocorticotropic hormone
BOLD: Blood oxygen level dependent
CSF: Cerebral spinal fluid
CV: Coefficient of variation
DEL: Deletion
FEW: Family-wise error
fT4: Free T4
GH: Growth hormone
HPA: Hypothalamic-pituitary-adrenal axis
ICC: Intraclass correlation coefficient
IGF-I: Insulin-like growth factor I
IR FSPGR: Inversion recovery fast spoiled gradient recalled
LOC: Lateral occipital complex
MNI: Montreal Neurological Institute
mUPD: Maternal uniparental disomy
PC: Posterior commissure
PPBS: Posterior pituitary bright spot
PVN: Paraventricular nucleus
PWS: Prader-Willi Syndrome
rs: Resting state
rT3: Reverse T3
SDS: Standard deviation score
TFCE: Threshold-free cluster enhancement.
